# Mapping the sensory perception of apple using descriptive sensory evaluation in a genome wide association study

**DOI:** 10.1371/journal.pone.0171710

**Published:** 2017-02-23

**Authors:** Beatrice Amyotte, Amy J. Bowen, Travis Banks, Istvan Rajcan, Daryl J. Somers

**Affiliations:** 1 Vineland Research and Innovation Centre, Victoria Avenue North, Vineland Station, ON, Canada; 2 University of Guelph, Department of Plant Agriculture, Guelph, ON, Canada; New South Wales Department of Primary Industries, AUSTRALIA

## Abstract

Breeding apples is a long-term endeavour and it is imperative that new cultivars are selected to have outstanding consumer appeal. This study has taken the approach of merging sensory science with genome wide association analyses in order to map the human perception of apple flavour and texture onto the apple genome. The goal was to identify genomic associations that could be used in breeding apples for improved fruit quality. A collection of 85 apple cultivars was examined over two years through descriptive sensory evaluation by a trained sensory panel. The trained sensory panel scored randomized sliced samples of each apple cultivar for seventeen taste, flavour and texture attributes using controlled sensory evaluation practices. In addition, the apple collection was subjected to genotyping by sequencing for marker discovery. A genome wide association analysis suggested significant genomic associations for several sensory traits including juiciness, crispness, mealiness and fresh green apple flavour. The findings include previously unreported genomic regions that could be used in apple breeding and suggest that similar sensory association mapping methods could be applied in other plants.

## Introduction

Apples (*Malus* x. *domestica*) are one of the most economically important temperate fruit crops in the world and one of the most diverse [1–2]. Apple breeders have been eager to exploit the broad genetic and phenotypic diversity of this species in order to meet consumer demands for new and delicious apples [3]. Research on existing cultivars suggest there is a strong consumer preference for apples with outstanding sweetness, crispness and juiciness [4–6]. These sensory fruit quality attributes present primary targets for consumer-focused plant breeding in apples.

Genomics approaches have been employed to define apple fruit quality at a genetic level, and some major causative genes have been found. For example, the control of fruit acidity by the *Ma* (Malic acid) locus on linkage group (LG) 16 was first mapped to a 150 kb region and then to a single nucleotide polymorphism (SNP) within the malate transporter-like gene *Ma1* [7–9]. Similarly, the control of apple fruit firmness (FF) measured by mechanical force was mapped to three quantitative trait loci (QTL) on LGs 1, 10 and 15 [10–12]. Subsequently, the genes *MdACO1* and *MdACS1* controlling climacteric ripening in apple fruit were found to lie within the texture QTL on LGs 10 and 15, respectively [13], and the expansin gene *MdExp7* involved in early fruit softening was positioned within the LG 1 QTL [14]. A polygalacturonase gene *MdPG1* also located on LG 10 was found to contain a SNP associated with FF [15]. These examples of confirmed fruit quality QTL represent a few of several loci for which marker assisted selection (MAS) is now routinely employed in apple breeding programs [16].

Conducting MAS for fruit quality traits promises to dramatically improve the efficiency of breeding apples [16]. Early selection for favourable alleles of the above-mentioned genes controlling apple acidity and firmness can enable breeders to rapidly enrich their programs for seedlings with favourable taste and texture. Both traits are critical targets for selection, as it is known that consumers prefer apples with firm, crisp texture and with a balance of sweet to acid taste [4–6]. Improvement in these and other quantitative fruit quality traits could be further achieved by the discovery and application of additional genetic markers for MAS. Given the decreasing cost of MAS and the relatively large cost of maintaining apple trees, ongoing investigations into the genetics of apple fruit quality are critical to continued gains in breeding efficiency [17–18].

New sources of variation for previously characterized traits including taste and texture may be uncovered by analysing more diverse apple germplasm in further QTL or association studies. A popular alternative to QTL mapping in simple or complex crosses is the genome-wide association study (GWAS) which does not require a designed mapping population [19–20]. Standard fruit quality traits were mapped in a six-parent family of 1200 apple seedlings as well as in a diverse collection of 115 apple accessions using GWAS [21–22]. Results from the two populations differed in terms of significant associations: titratable acidity (TA) was mapped to LG 8 in the seedling population versus LG 16 in the diversity collection. While both significant loci mapped to LGs at which QTL for TA had previously been reported [12,23], the results of these studies demonstrate that mapping in diverse germplasm can reveal distinct loci from those found in segregating populations. The loci detected through both multi-family and diverse germplasm mapping studies have broad practical application for breeding, as they capture QTL present in numerous potential breeding parents. A specific advantage to GWAS of diverse germplasm is that it permits finer resolution of QTL. Historical recombination events have reduced the extent of linkage disequilibrium (LD) within diverse germplasm, therefore significant markers are more likely to be physically proximal to the true genetic regions controlling variation in fruit quality [19].

New sources of variation in fruit quality may also be revealed by using alternative phenotyping approaches. For example, significant efforts have been made to characterize the genetic components of apple texture through instrumental analyses. Crisp apple texture results from the breaking of apple cell walls through biting and mastication, while mealy apple texture results from the separation of cells at the middle lamella without breaking [24]. Texture phenotyping has predominantly consisted of measuring fruit firmness as the mechanical force required to penetrate an apple with a blunt probe [10]. However when the components of apple texture were broken down into four acoustic and eight mechanical parameters, significant QTL were discovered on all but four of the 17 apple LGs, including both new and previously reported loci [25]. A similarly large number of texture QTL were detected in a separate study when mechanical compression and penetration parameters were mapped to 14 LGs alongside sensory descriptors of apple texture [26]. These studies illustrate the genetic complexity of apple texture and the potential for new QTL to be discovered by mapping fruit quality traits with alternative phenotyping approaches.

Descriptive sensory evaluation of apples by a trained sensory panel is yet another means by which phenotypic fruit quality data can be generated for QTL discovery. Descriptive sensory evaluation involves the use of an independent panel of highly trained sensory analysts who are able to consistently quantify unique taste, texture and flavour attributes in apple fruits [27–28]. Each member of the sensory panel defines the intensity of pre-determined fruit quality traits in samples of apple, while blind to the types of apple and the sampling order [6,28].

The potential advantage of descriptive sensory evaluation for QTL discovery lies in its applicability to consumer focused plant breeding [4]. Most apple genetic mapping studies for fruit quality use instrumental measures to model the human perception of apple taste, flavour and texture. However some apple traits evaluated by trained sensory analysts can differ significantly from instrumental predictions [29–30]. Instrumental fruit firmness was found to be weakly correlated with sensory crispness (r = 0.5) and uncorrelated with juiciness (r = -0.2) in 115 segregating seedlings of ‘Prima’ x ‘Fiesta’ [10]. Similar weak correlations were observed between the same instrumental and sensory traits (r < 0.3) in 33 advanced apple selections and cultivars [31]. TA was found to be highly correlated with acid apple taste in 15 segregating seedlings and parental cultivars assessed by sensory panelists (r = 0.9), but sweet taste could not be predicted by either sugar concentration (r = 0) or soluble solids content (SSC) (r = 0.4) [32]. Thus, using trained sensory measures to map fruit quality traits may provide insight into important additional QTL that are not captured by standard instrumental analyses.

Despite its potential, descriptive sensory evaluation for QTL discovery in apple is limited by high cost and low throughput. Both the cost of employing the panel and the length of time required for the evaluation considerably restrict the number of apple genotypes that can be included in such a study. A sensory panel is ideally composed of ten to twenty independent analysts who are able to precisely and repeatedly detect and describe taste, flavour and texture attributes of food products [27–28]. The panel must be trained over several weeks to develop a list of descriptors and define intensity ranges for each quality attribute of the food product. During the formal evaluation period, analysts must evaluate each sample for every attribute previously defined by the panel [27–28]. Controlled methods are applied to avoid common confounding effects in fruit tasting including name recognition bias, sample order bias, trait interaction and panelist fatigue [27,33]. The results are quantitative and repeatable measures of fruit quality traits as perceived by human consumers [6,28].

Continued advancement in apple breeding relies critically upon the discovery of new genomic targets for MAS; such advancement can be sought both through the assessment of distinct apple populations as well as through alternative approaches to phenotypic data collection. In this study we employed a trained sensory panel to evaluate a moderately sized and diverse collection of apple cultivars over two years. We then used genotyping by sequencing (GBS) [34] data to conduct a GWAS of apple sensory taste, flavour and texture. Here we show that through descriptive sensory evaluation we can identify fruit quality loci which may have direct application in breeding fruit for consumer preference. This study demonstrates the applicability of combining sensory science with genomic analysis to define quality in horticulture products.

## Materials and methods

### Apple germplasm

The germplasm used in this study consisted of 85 apple cultivars which included 57 heritage cultivars and 28 commercial cultivars (Table 1). This population was chosen to best capture the available variation in apple breeding germplasm while maintaining a feasible population size for descriptive sensory evaluation. The heritage cultivars were selected from a collection at the Vineland Research and Innovation in Vineland, Ontario, Canada, based on reports of their distinct fruit quality attributes and diverse origins [35–36]. The commercial cultivars were selected based on their availability from Canadian commercial apple growers and retailers.

**Table 1:** A list of 85 apple cultivars used in the study. Each apple is designated either commercial or heritage, red (R) or green (G) and a three-letter cultivar code is given.

Apple Cultivar	Designation	Skin Colour	Code
Ambrosia	Commercial	R	AMB
Aurora Golden Gala^TM^	Commercial	G	AUR
Cortland	Commercial	R	COR
Creston	Commercial	G	CRE
CrimsonCrisp^TM^	Commercial	R	CRI
Divine^TM^	Commercial	R	DIV
Elstar (Commercial)	Commercial	R	ELC
Empire	Commercial	R	EMP
Fuji	Commercial	R	FUJ
Ginger Gold	Commercial	G	GIN
Golden Delicious	Commercial	G	GOD^A^
Granny Smith	Commercial	G	GRA
Honeycrisp	Commercial	R	HON
Idared	Commercial	R	IDA
Jazz^TM^	Commercial	R	JAZ
McIntosh	Commercial	R	MCI
Mutsu	Commercial	G	MUT^A^
Nicola^TM^	Commercial	R	NIC
Northern Spy	Commercial	R	NOS^A^
Pink Lady^TM^	Commercial	R	PIN
Red Delicious	Commercial	R	RED
Red Prince	Commercial	R	REP
Royal Gala	Commercial	R	ROY
Salish^TM^	Commercial	R	SAL
Silken	Commercial	G	SIL
Smitten	Commercial	R	SMI
SweeTango^TM^	Commercial	R	SWE
Tentation	Commercial	G	TEN
Akane	Heritage	R	AKA
Antonovka	Heritage	G	ANT
Baldwin	Heritage	R	BAL
Blenheim Orange	Heritage	G	BLE
Blue Pearmain	Heritage	R	BLU
Bottle Greening	Heritage	G	BOT
Canada Red	Heritage	R	CAN
Chenango Strawberry	Heritage	R	CHE
Colvert	Heritage	G	COL
Cox's Orange Pippin	Heritage	R	COX
Dawn Mac	Heritage	R	DAW
Early Joe	Heritage	G	EAR
Elstar (Heritage)	Heritage	R	ELH
Esopus Spitzenburg	Heritage	R	SPI
Fameuse	Heritage	R	FAM
Freedom	Heritage	R	FRE
Golden Russet	Heritage	G	GOL
Grimes Golden	Heritage	G	GRI
Haas	Heritage	R	HAA
Heritage Gala	Heritage	R	GAH^A^
Hume	Heritage	R	HUM
Irish Peach	Heritage	G	IRI
Jersey Mac	Heritage	R	JER
Jonathan	Heritage	R	JON^A^
King	Heritage	R	KIN
Leder Borsdorf	Heritage	R	LED
Liberty	Heritage	R	LIB
Lobo	Heritage	R	LOB
Lodi	Heritage	G	LOD
Lubsk Queen	Heritage	G	LUB
Macoun	Heritage	R	MAC
McMahon	Heritage	G	MCM
Moscow Pear	Heritage	G	MOS
Moyer Heritage	Heritage	G	MOY
Newtown Pippin	Heritage	G	NEW
North Star	Heritage	R	NOR
NovaSpy	Heritage	R	NOV^A^
Ontario	Heritage	G	ONT
Pear Gold	Heritage	G	PEA
Pomme Grise	Heritage	G	POM
Quinte	Heritage	R	QUI
Red Atlas	Heritage	R	REA
Rome Beauty	Heritage	R	ROM
Roxbury Russet	Heritage	R	ROX
Russet	Heritage	G	RUS^A^
Snow	Heritage	R	SNO
Spartan	Heritage	R	SPA
St Lawrence	Heritage	G	STL
Summer Rambo	Heritage	R	RAM
Tolman Sweet	Heritage	G	TOL
Vinebrite	Heritage	R	VIN
Vista Bella	Heritage	R	VIS
Wealthy	Heritage	R	WEA
White Winter Calville	Heritage	G	WHI
Winter Banana	Heritage	G	WIN
Yellow Bellflower	Heritage	G	YEB
Yellow Transparent	Heritage	G	YET

^A^ Cultivars used only for SNP discovery (not phenotyped).

### Apple genotyping

Genomic DNA was extracted from leaf tissue from individual clones of each apple cultivar and subjected to GBS [34]. DNA samples were sent in duplicate to the Genomic Diversity Service at Cornell University for GBS library preparation using the restriction enzyme *Ape*KI and sequencing using an Illumina HiSeq 2000 instrument (Illumina Inc., San Diego, CA). Raw sequencing reads were processed with the TASSEL-GBS pipeline [37] using default parameters and calling heterozygous genotypes based on the reference genome *Malus* x *domestica* v3.0.a1 [1,38].

The apple SNP data were stringently filtered using TASSEL 5.2 [39] to improve the density and distribution of allele calls [40]. SNP loci with greater than 5% missing data were removed and the remaining missing allele calls were imputed using the default parameters of linkage disequilibrium-k nearest neighbor imputation [41]. Following imputation, a stringent 10% minor allele frequency (MAF) filter was applied to yield the SNP data set used in all downstream analyses. The SNP polymorphism data is provided (S5 Table) and all sequencing reads were submitted to the NCBI Short Read Archive (SRA) under the accession number SRX2437486.

### Apple population statistics

Population structure and LD were evaluated based on variation in all SNP markers. Kinship and population stratification were evaluated in TASSEL 5.2 [39] using the Centered-Identity by State (Centered-IBS) [42] and Principal Component Analysis (PCA) methods, respectively. Pairwise LD between all SNP pairs on a chromosome was estimated as r^2^ using the software PLINK 1.07 [43] and the chromosome-wise LD decay was estimated at a critical r^2^ value of 0.2 [44].

### Apple sensory and instrumental evaluation

Phenotypic evaluations were conducted in 2012 and 2013. Each genotype from the apple population used in this study was designated as “heritage” or “commercial” based on the source of fruit (Table 1). The heritage apples were harvested from a small orchard located onsite at the Vineland Research and Innovation Centre. Heritage apple maturity was determined by visual inspection, tasting and a starch-iodine (SI) index rating of five or higher according to the Cornell scale [45]. The commercial apples were sourced directly from Canadian apple growers or purchased from Canadian grocery retailers.

Both heritage and commercial apples were placed in cold storage (2°C; high humidity; normal atmosphere) for 10 to 20 days prior to phenotypic evaluation. This range of storage times was chosen to best fit the sensory panel schedule which could accommodate up to six apple cultivars per weekly session. The heritage apple cultivars were evaluated as close as possible to their harvest SI indices while commercial apple cultivars were evaluated at SI indices of five or higher. Apples were removed from cold storage and placed at room temperature for 24 hours prior to evaluation. Due to challenges in fruit production and timing, seven apple cultivars were not included in the phenotypic evaluation (Table 1).

Descriptive sensory evaluation of the apple cultivars was conducted using a panel of 20 trained sensory analysts. Each analyst had previously been selected from a pool of 100 applicants screened for their ability to precisely discern and describe sensory attributes in food products [27]. For this study, the sensory panel first developed a set of sensory descriptors for apple taste, flavour and texture attributes using a consensus method previously applied in descriptive sensory evaluation of apple [5,28].

The formal descriptive sensory evaluations were conducted in a dedicated sensory laboratory equipped with isolation booths, red lights to disguise apple skin colour and physical separation from the sample preparation area [27–28]. The sensory panel examined four to six apple cultivars per session which included two replicates of each cultivar, presented in a randomized order. The number of cultivars per session was limited to avoid panel fatigue and to ensure continued accuracy over time [33]. The panel was blind to both the purpose of the study and the component apple cultivars. The sensory panel scored each apple sample for seven texture traits, seven flavour traits and three taste traits. Each sensory trait was scored on a scale of zero to 100, from no perceived intensity to the highest perceivable intensity. A short definition of each sensory attribute is given in Table 2.

**Table 2:** Description of quantitative apple fruit quality traits. Instrumental traits were defined and measured using standard methods as indicated. Sensory traits were defined and measured using descriptive sensory evaluation by a trained panel of 20 sensory analysts.

Trait Type	Trait^A^	Definition
Instrumental	Flesh Firmness	mechanical force required to penetrate peeled apple flesh
	Soluble Solids Content	soluble solids content in apple juice
	Titratable Acidity	titratable acidity in apple juice
Sensory Taste	Acid	acidic taste in apple flesh and juice
	Bitter	bitter taste in apple flesh and juice
	Sweet	sweet taste in apple flesh and juice
Sensory Flavour	Earthy	earthy, musty aroma
	Floral	floral aroma
	Fresh Green Apple	grassy, vegetal aroma
	Fresh Red Apple	apple aroma
	Honey	honey aroma
	Lemony	lemony aroma
	Oxidized Red Apple	oxidized apple aroma
Sensory Texture	Astringent	sensation of dry, puckering mouthfeel in apple flesh and juice
	Chewy	amount and duration of chewing movements needed to rend apple flesh
	Crisp	sound and sensation of breaking apart apple flesh in a single bite
	Juicy	amount of liquid released from apple flesh by chewing
	Mealy	sensation of soft, granular apple flesh
	Rate of Melt	amount of apple flesh melted after a certain number of chews
	Skin Thickness	amount of force needed to bite through apple skin

^A^ Traits were recorded in the following units: Flesh Firmness (kg), Titratable Acidity (g malic acid L^-1^), Soluble Solids Content (% Brix), Sensory Traits (perceived intensity: 0 to 100).

Standard instrumental fruit quality data were collected on the same day as sensory evaluations for each cultivar (Table 2). Skin colour was recorded as presence or absence of red colouration [40]. FF was evaluated for five apples of each cultivar using a TA-XT Plus texture analyzer (Texture Technologies, Hamilton, MA). Texture analysis was conducted on two opposite, peeled sides of each apple, and FF was recorded as the maximum force (kg) applied by a blunt 11 mm diameter probe at 4.5 mm s^-1^ with an 8 mm penetration depth [46]. SSC and TA were measured in duplicate from three independent juice samples of each cultivar. SSC (% Brix) of each juice sample was measured using a digital refractometer. TA (g malic acid L^-1^ juice) was measured by titrating 2 mL of each juice sample to pH 8.1 with 0.1 N sodium hydroxide [8].

### Apple genome-wide association study

Best linear unbiased predictors (BLUPs) of apple cultivar (genotype) effects were estimated for each of the phenotypic fruit quality traits. Genotypic BLUPs were estimated through a restricted maximum likelihood mixed linear variance analysis [47] using the lmer procedure of the lme4 package [48] in R 3.2 [49]. Broad sense trait heritabilities were estimated from the restricted maximum likelihood variance components [50]. Genotypes were also extracted as BLUPs for single and combined years to evaluate year-to-year consistency for associations. All trait BLUPs were adjusted with their trait means in order to be expressed on a practical scale for downstream analyses. Pearson correlations between years and Spearman rank correlations between traits were estimated from adjusted BLUPs using the rcorr procedure of the Hmisc package [51] in R 3.2 [49].

Associations between the apple GBS data and trait BLUP values were evaluated in TASSEL 5.2 [39]. Six principal components (PCs) were included based on a scree test [52] to account for effects of population structure in both general linear model (GLM) and mixed linear model (MLM) analyses. The Centered-IBS matrix [42] was included to account for kinship effects in the MLM and default parameters were used for both compression and variance component estimation. A 5% false discovery rate (FDR) significance threshold for each association study was assigned using the Benjamini-Hochberg FDR correction [53]. In addition to conducting association studies for all quantitative fruit quality traits, the bimodal skin colour trait (red vs. yellow or green) was included as a reference phenotype to test the integrity of the SNP data and association models. Manhattan plots for association mapping results were visualized using the qqman package [54] in R 3.2 [49].

Loci with consistent effects for fruit quality traits across GWAS models were examined individually for segregation of SNP haplotypes and associated phenotypes. The effects of SNP allele on trait value were evaluated using a simple variance analysis in SAS 9.3 (SAS Institute, Cary, NC) for loci with mostly balanced haplotype distributions. Potential gains from selection were estimated as the difference between the mean of the favourable allele classes and the trait mean, and reported as a percent of the trait mean.

Genomic regions with significant SNPs were queried for putative candidate genes associated with apple fruit quality. Localized LD within the 0.5 Mb surrounding the locus of interest was estimated in PLINK1.07 [43] and plotted using the ggplot2 [55] package in R 3.2 [49]. A list of genes positioned within 0.5 Mb of the locus of interest was obtained for the *Malus* x *domestica* v3.0.a1 reference genome [1] from the Genome Database for Rosaceae [38] with gene ontology annotations and the first alignment of a protein BLAST [56]. The gene list was filtered to retain genes with non-redundant names, sequences and approximate positions. Filtered gene locations were plotted based on approximate start positions without scaling.

## Results

### Genotyping by sequencing

The GBS data set for 85 apple genotypes was generated from 490,753,997 raw Illumina sequencing reads. These data yielded 191,762 polymorphic SNP loci with 15 to 75% missing data per genotype and 20% missing data overall. The SNP data were first filtered to retain 76,492 SNPs with less than 5% missing data per locus. The data set was then imputed [41] and further filtered to a minimum 10% MAF. The final SNP set had 0.35% missing data overall.

The final GBS marker set had 52,440 polymorphic SNPs with an average of 3,085 SNPs per chromosome (S1 Table). The 52,440 SNPs provided a genome-wide coverage of 1 SNP per 14.2 kb of the 742.3 Mb apple genome, and saturating coverage for all chromosomes based on the extent of LD. The extent of LD across each chromosome was estimated at a critical r^2^ value of 0.2 [44] and found to extend an average distance of 58.4 kb (S1 Table).

### Population structure

**Fig 1:**
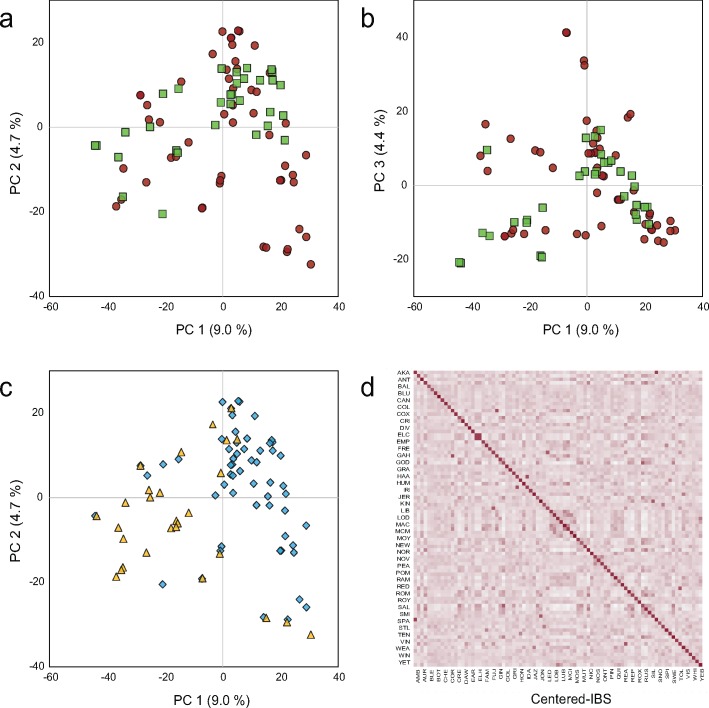
Population structure of 85 apple cultivars based on genotypic variation. Principal component analysis biplots show (a,b) red apple cultivars represented by red circles and green/yellow cultivars represented with green squares, or (c) commercial apple cultivars represented by yellow triangles and heritage apple cultivars represented by blue squares. (d) Centered identity-by-state kinship heat map show close pairwise relatedness represented by dark values and distant relatedness represented by light values. Cultivar codes are defined in Table 1.

The 85 apple cultivars analyzed in this study showed a high level of genetic diversity with little population structure (Fig 1, Table 1). A PCA revealed a low degree of population stratification, with the first three PCs accounting for 18% of the population-wide SNP variation. No genomic distinctions between apples of red and yellow or green skin colour were observed, however there was some separation across the first two PCs between the commercial and heritage apple cultivars. The Centered-IBS kinship analysis [42] also revealed an overall low level of relatedness (range -0.2–0.98; average IBS < 0) with very few pairs of closely related genotypes within the apple population (Fig 1).

### Phenotypic variation

**Fig 2:**
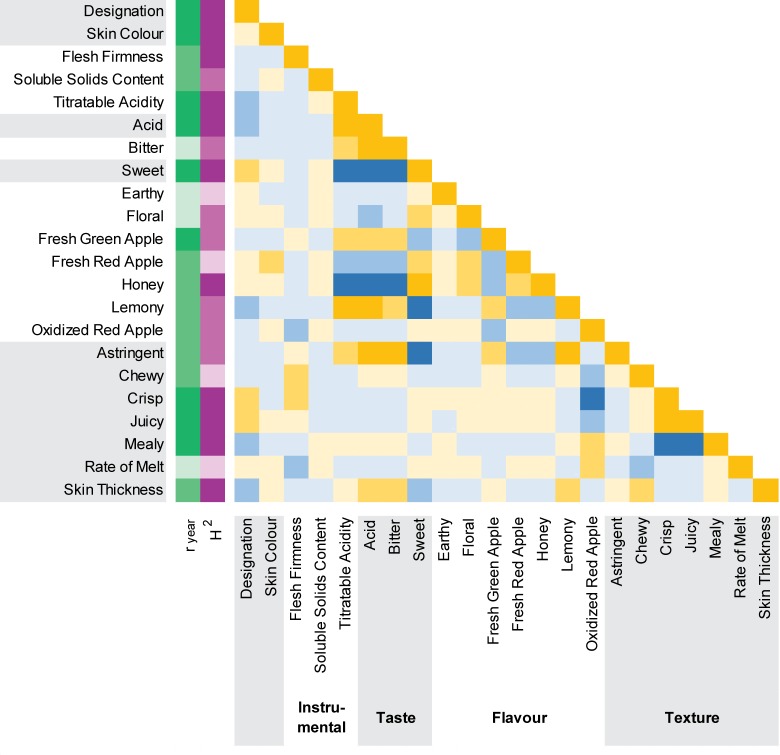
Year to year correlations, heritabilities and pairwise correlations for apple fruit quality traits. “Designation” refers to apple cultivars as either commercial or heritage, “r_year_” is the Pearson correlation between years for each trait (green colour intensity corresponds to r_year_ magnitude), and “H^2^” is the broad sense trait heritability (violet colour intensity corresponds to H^2^ magnitude). Spearman rank correlations between traits range from highly positive (dark gold) to highly negative (dark blue).

The phenotypic variation in 20 sensory and instrumental fruit quality traits was evaluated in 2012 and 2013 (S2 Table). Since adequate fruits could not be obtained for seven cultivars, phenotyping was conducted for 78 of the 85 apple cultivars (Table 1). The year to year Pearson correlations (r_year_) were moderate and the broad sense trait heritabilities (H^2^) were high for the three instrumental traits which included FF, SSC and TA (Fig 2). Both r_year_ and H^2^ were more variable for the 17 sensory traits which included three taste attributes, seven flavour attributes and seven texture attributes. The sensory traits with the highest r_year_ and H^2^ values included *acid* and *sweet* taste, *fresh green apple* flavour, and *crisp*, *juicy* and *mealy* texture (Fig 2).

Pairwise Spearman rank correlations (ρ) were very weak between the instrumental fruit quality traits and their related sensory traits, except in the case of TA (Fig 2, S1 Fig). TA was positively correlated with *acid* (ρ = 0.85) and negatively correlated with *sweet* taste (ρ = -0.76). Several sensory traits were positively correlated with TA and *acid* taste including *bitter* taste, *fresh green apple* and *lemony* flavour, and *astringent* texture. As well, several sensory traits were negatively correlated with TA and *acid* taste, but positively correlated with *sweet* taste including *floral*, *fresh red apple* and *honey* flavour. SSC was poorly correlated with *sweet* taste (ρ = 0.18) (Fig 2).

Sensory texture was weakly correlated with FF, with the absolute value of ρ below 0.5 for FF compared with *astringent*, *chewy*, *crisp*, *juicy*, *mealy* and *skin thickness* attributes (Fig 2, S1 Fig). Among sensory texture traits there was a positive correlation between *juicy* and *crisp* (ρ = 0.79). Both *juicy* and *crisp* were also negatively correlated with *mealy* texture (ρ < -0.80). The remaining sensory texture traits including *skin thickness* and *rate of melt* were poorly correlated with *juicy*, *crisp* and *mealy* texture (Fig 2).

### Genome-wide association study

**Fig 3:**
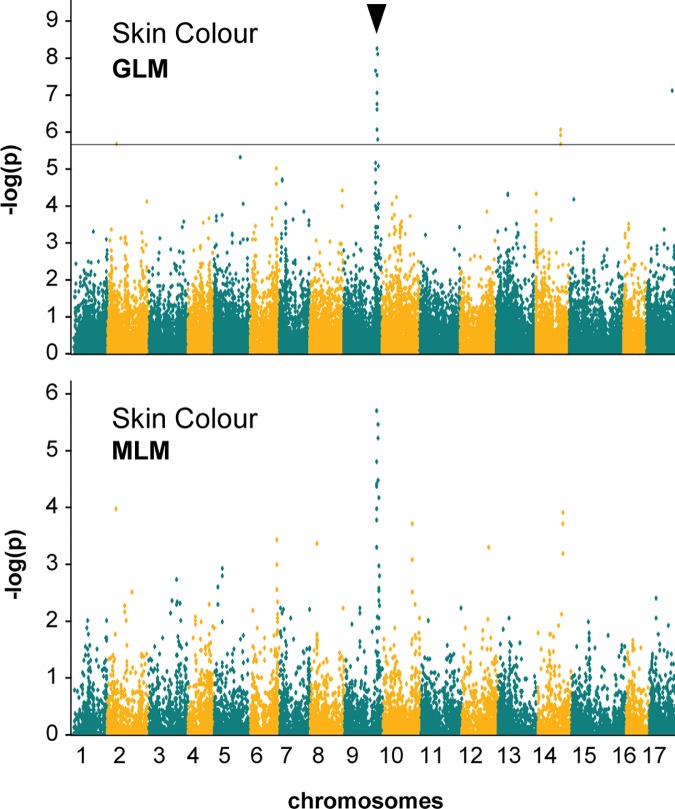
Manhattan plots for the apple reference trait ‘skin colour’ in 78 apple cultivars using GLM and MLM analyses. The most significant SNPs are indicated with black arrows; the 5% false discovery rate is indicated by the horizontal line.

The SNP data were tested for reliability in GWAS using a trait with a known genomic position prior to analysis of quantitative fruit quality traits. Apple skin colour (red vs. yellow or green) was mapped to chromosome 9 with the most significant SNP at position Chr9:29,904,993 (Fig 3). This apple skin colour locus corresponded with the physical position of the *MdMYB1* (also known as *MdMYB10*) gene controlling red skin colour [38,57]. The chromosome 9 skin colour locus was significant in the GLM analysis which included the fixed effects of population stratification but not the random effects of kinship [39,58]. Although it did not reach the 5% FDR threshold of significance, the Manhattan plot showed a clear association for the chromosome 9 locus in the MLM analysis which included both population stratification and kinship effects [39,58]. The apple skin colour SNP peaks observed in the MLM analysis were coincident with those detected in the GLM analysis (Fig 3).

**Fig 4:**
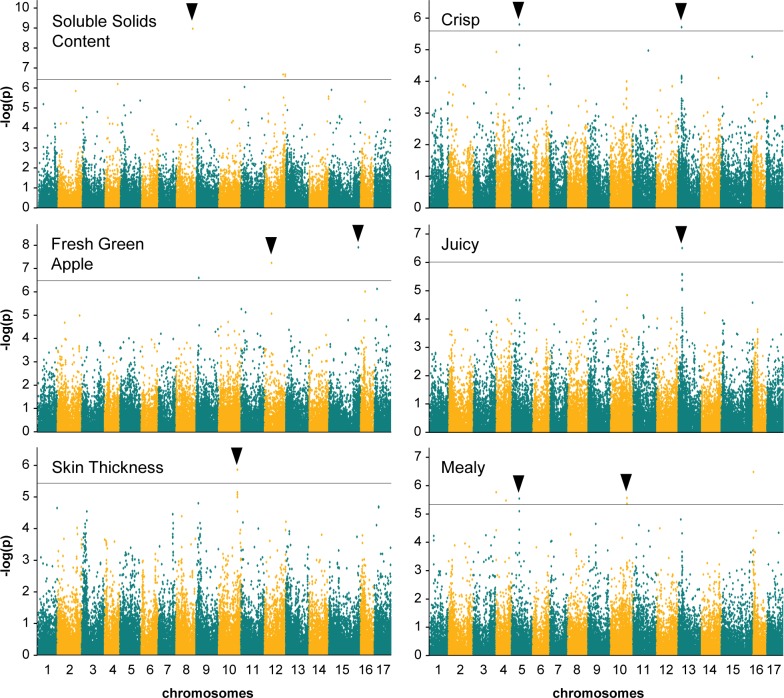
Manhattan plots for six fruit quality traits in 78 apple cultivars using GLM. Instrumental traits include SSC. Sensory traits include *fresh green apple*, *skin thickness*, *juicy*, *crisp* and *mealy*. The most significant SNPs with repeated effects are indicated with black arrows; the 5% FDR is indicated by the horizontal line in each panel.

The same SNP data and analysis methods were used to conduct a GWAS of 20 quantitative fruit quality traits with genotypic BLUPs estimated from 2012, 2013 and combined year data. Significant marker effects were found for nine of the 20 traits evaluated using GLM analyses. However only six traits had markers with repeated effects over two years (Fig 4, Table 3). These traits included instrumental SSC, the sensory flavour attribute *fresh green apple*, and the sensory texture attributes *crisp*, *juicy*, *mealy* and *skin thickness*. The SNPs with the highest significance in GLM were reported for all loci with repeated effects. The MLM analysis did not detect any significant SNP effects for any of the quantitative fruit quality traits (Fig 4, Table 3).

**Table 3:** Association statistics of loci most significantly associated with apple fruit quality traits in combined years. GLM statistics are given for single and combined year data; MLM statistics are given for combined year data.

Trait	SNP Position	2012 (GLM)			2013 (GLM)			Combined Years (GLM)			Combined Years (MLM)	
		p-value		R^2^	p-value		R^2^	p-value		R^2^	p-value	R^2^
SSC	Chr8:24,235,959	2.6 x 10^−4^		0.24	1.4 x 10^−8^	*	0.35	3.1 x 10^−10^	*	0.43	5.5 x 10^−4^	0.22
Fresh Green Apple	Chr9:4,092,040	4.9 x 10^−6^		0.24	8.3 x 10^−5^	*	0.20	1.8 x 10^−7^	*	0.26	1.2 x 10^−4^	0.26
	Chr12:10,115,943	3.0 x 10^−7^	*	0.28	1.2 x 10^−5^	*	0.23	4.0 x 10^−8^	*	0.28	6.4 x 10^−4^	0.22
Crisp	Chr5:11,526,314	1.0 x 10^−6^	*	0.23	2.1 x 10^−6^		0.24	1.0 x 10^−6^	*	0.23	5.2 x 10^−5^	0.31
	Chr13:6,049,060	3.8 x 10^−4^		0.17	6.8 x 10^−6^		0.22	1.3 x 10^−6^	*	0.23	1.0 x 10^−5^	0.37
Juicy	Chr13:6,049,060	4.2 x 10^−4^		0.15	7.0 x 10^−7^	*	0.25	3.7 x 10^−7^	*	0.25	8.5 x 10^−5^	0.27
Mealy	Chr5:11,526,314	2.0 x 10^−9^	*	0.34	3.7 x 10^−4^		0.16	3.0 x 10^−6^	*	0.23	9.3 x 10^−5^	0.29
	Chr10:24,387,143	1.5 x 10^−4^		0.19	1.2 x 10^−5^	*	0.19	4.7 x 10^−6^	*	0.26	1.5 x 10^−3^	0.19
Skin Thickness	Chr10:28,123,441	1.7 x 10^−2^		0.10	3.5 x 10^−7^	*	0.29	9.6 x 10^−7^	*	0.25	1.0 x 10^−4^	0.28

* Marker-trait association significant based on a 5% Benjamini-Hochberg FDR (Benjamini and Hochberg 1995).

A significant locus for SSC was detected on chromosome 8 for 2013 and combined year data (Fig 4, Table 3). The range of values for SSC within the population was 12 to 17% Brix (S2 Table). The GLM GWAS results indicated that the chromosome 8 locus (Chr8:24,235,959) explained 43% (R^2^ = 0.43) of variation in SSC for combined years (Table 3). No significant marker effects were detected for sensory *sweet* taste, which was poorly correlated with SSC (Fig 2).

Two significant loci for *fresh green apple* flavour were detected on chromosomes 9 and 12 for 2012 and combined year data (Fig 4, Table 3). The range of values for *fresh green apple* flavour intensity within the population was 12 to 22 points (S2 Table). The intensity of *fresh green apple* flavour did not correspond to the colour of the apple cultivars (ρ = -0.26) (Fig 2). The GLM GWAS indicated that the chromosome 9 (Chr9:4,092,040) and chromosome 12 (Chr12:10,115,943) loci respectively explained 26% and 28% of the variation in *fresh green apple* flavour for combined years (Table 3). No significant marker effects were detected through GWAS for TA or for the sensory taste attribute *acid*, both of which were positively correlated with *fresh green apple* flavour (Fig 2).

Two significant loci were shared by the correlated texture traits *crisp*, *juicy* and *mealy* (Fig 4, Table 3). The range of texture intensity values was 16 to 59 points for *crisp*, 22 to 57 points for *juicy* and 14 to 53 points for *mealy* (S2 Table). A significant locus on chromosome 5 was found for both *crisp* and *mealy* texture in 2012 and combined years. GLM GWAS results indicated that the chromosome 5 locus (Chr5: 11,115,943) explained 23% of the variation in both *crisp* and *mealy* texture for combined years. A second significant locus was detected on chromosome 13 for *crisp* texture in combined years and for *juicy* texture in 2013 and combined years. GLM GWAS results showed that the chromosome 13 locus (Chr13:6,049,060) explained 23% of the variation in *crisp* and 25% of the variation *juicy* texture for combined year data (Table 3).

Two additional significant loci for apple sensory texture were detected on chromosome 10 (Fig 4, Table 3). A significant locus on chromosome 10 was found for *mealy* texture in 2013 and combined years. GLM GWAS results indicated that this locus (Chr10:24,387,143) explained 26% of the variation in *mealy* texture for combined years. Another significant locus on chromosome 10 was also identified for *skin thickness* for 2013 and combined years (Fig 4, Table 3). *Skin thickness* was poorly correlated with *mealy* texture (ρ = 0.26, Fig 2). The range of intensity values for *skin thickness* within the population was 38 to 52 points (S2 Table). The GLM GWAS estimated that this locus (Chr10:28,123,441) explained 25% of the variation in *skin thickness* for combined year data (Table 3). No additional significant loci were detected for apple texture traits, including instrumental FF.

### Candidate sensory trait loci

**Fig 5:**
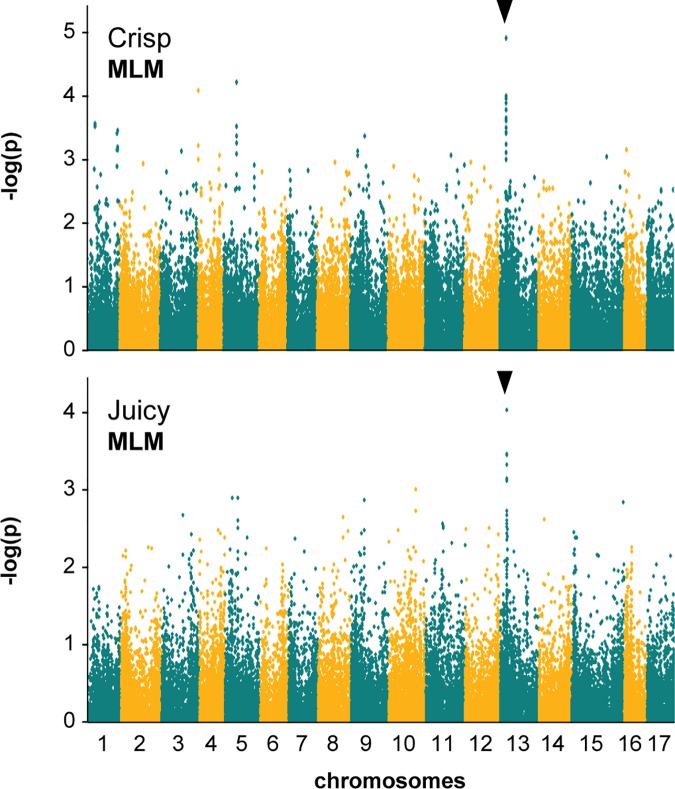
Manhattan plots for crisp and juicy sensory texture in 78 apple cultivars using MLM. The locus of interest is indicated with open arrows; all SNPs are below the 5% FDR threshold.

The significant associations detected in GLM GWAS were compared against MLM analyses. SNP peaks which corresponded with the significant GLM loci were not observed in the MLM Manhattan plots for the traits SSC, *fresh green apple*, *mealy* or *skin thickness* (Table 3). However the MLM analyses for *crisp* and *juicy* texture did show a peak of SNP markers on chromosome 13 that were coincident with the significant locus detected in GLM analyses (Table 3, Fig 5).

**Fig 6:**
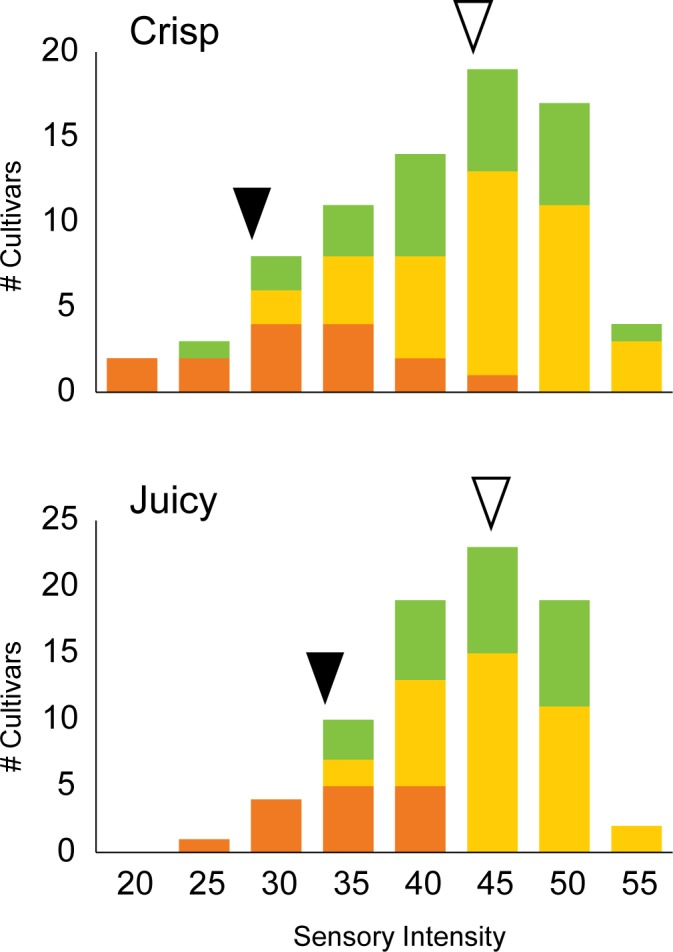
Stacked frequency distribution of 78 apple cultivars for crisp and juicy sensory texture based on allele class at Chr13:6,049,060. The mean trait values of the favourable AA (green) and AC (orange) alleles are indicated with open arrows and the means of the non-favourable CC (red) alleles are indicated with closed arrows. Sensory intensity values are scored on a scale of zero to 100.

The Chr13:6,049,060 sensory texture locus was examined in a simple variance analysis which considered the effects of allele class on apple texture (Fig 6, S3 Table). The results of this analysis indicated that allele class at Chr13:6,049,060 explained 34% of the variation in *crisp* and 40% of the variation in *juicy* texture. The mean difference in texture intensity between homozygous allele classes was 13.4 points for *crisp* and 11.5 points for *juicy* (Fig 6). A significant effect of allele class at Chr13:6,049,060 was also detected for *mealy* texture (p < 0.0001) which explained 29% of the trait variation (S3 Table). *Mealy* texture was strongly correlated with *crisp* and *juicy* attributes (Fig 2). The allele class at this locus had a small effect on the instrumental texture trait FF (p < 0.05), but FF was poorly correlated with *crisp*, *juicy* and *mealy* sensory texture (Fig 2, S3 Table).

The Chr13:6,049,060 sensory texture locus had a balanced distribution of alleles with 15 homozygous unfavourable, 38 heterozygous and 25 homozygous favourable individuals (Fig 6). The homozygous unfavourable allele class represented low *crisp*, low *juicy* and high *mealy* trait values. There were no significant differences between the homozygous favourable and heterozygous allele classes which together represented high *crisp*, high *juicy* and low *mealy* trait values (Fig 6, S3 Table). The homozygous favourable allele class included commercial cultivars such as ‘Ambrosia,’ ‘Honeycrisp’ and ‘Jazz’. The heterozygous allele class included commercial cultivars such as ‘Fuji,’ ‘Silken’ and ‘SweeTango.’ The homozygous unfavourable allele class included the commercial cultivar ‘McIntosh’ as well as the heritage cultivars ‘Blue Pearmain’ and ‘Lodi.’

**Fig 7:**
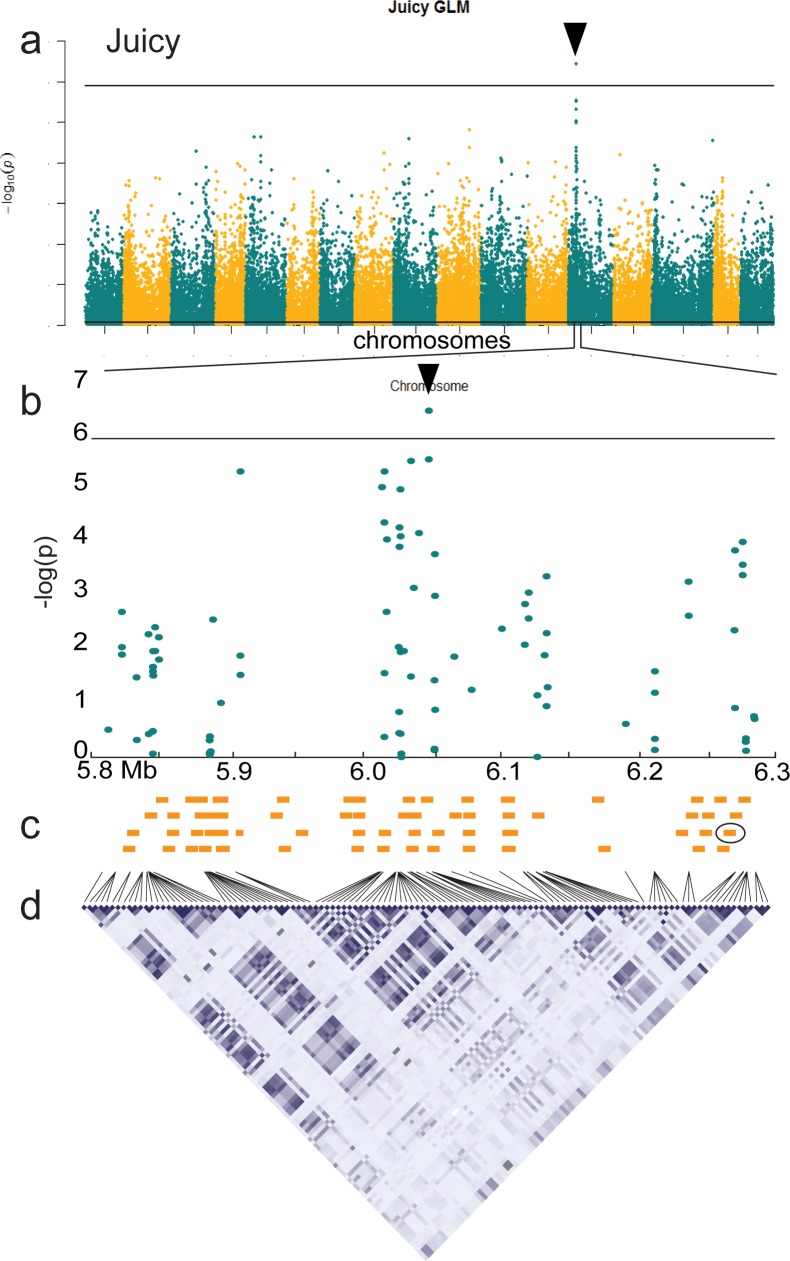
Detailed view of the apple sensory texture locus on chromosome 13. (a) Manhattan plot for *juicy* texture with (b) a detailed view of Chr13: 5.8 Mb to 6.3 Mb showing the significant association at Chr13:6,040,960. (c) Non-redundant *Malus* x *domestica* gene positions (not to scale) with MDP0000219907 circled. (d) Physical positions and LD for the 122 SNPs in the 0.5 Mb region (colour intensity corresponds with r^2^ magnitude).

The genomic region surrounding Chr13:6,049,060 was examined for candidate genes associated with textural fruit quality. The average LD on chromosome 13 extended 122 kb, and a region of high localized LD was observed from 5.8 to 6.1 Mb (Fig 7, S1 Table). There were 68 putative non-redundant genes within the 500 kb bordering the significant texture locus. The approximate locations for each of these genes based on start position are presented (Fig 7). The nine genes most proximal to the texture locus (within 50 kb) included one putative cytochrome p450, one vacuolar membrane protein and seven genes of unknown function. A putative pectin methylesterase (PME) gene (MDP0000219907) potentially involved in regulating the structure and breakdown of apple fruit cell walls was located at Chr13:6,262,833, approximately 214 kb from the sensory texture locus (Fig 7) [38,59–60]

## Discussion

The present GWAS made use of a moderately sized, diverse population of 85 commercial and heritage apple cultivars with a wide range of fruit quality attributes (Fig 1, S2 Table). The population size was limited in order to accommodate the high cost and low throughput nature of descriptive sensory evaluation [4,28,61] and facilitated the detection of large marker effects with sufficient statistical power (Figs 3 and 4) [19–20]. The GBS SNP data showed a high level of population diversity with very little structure (Fig 1). The use of descriptive sensory phenotyping combined with the diverse population and robust GBS data enabled the discovery of previously unreported apple fruit quality loci including one novel locus associated with juiciness and crispness which are both primary drivers of consumer preference (Figs 4–7) [4–6].

### Apple population

The apple population featured a high level of genotypic diversity (Fig 1). Previous apple GWAS have reported genomic population structure with subpopulations arising from distinct geographical origins [22] or from distinct market classes [62]. A large GWAS of 1200 diverse peach accessions also reported a clustering of subpopulations according to geographical origin, with more than 20% of the population structure captured in the first two PCs [63]. In our study, the first three PCs captured only 18% of the genomic population structure and did not show any sub-clusters of genotypes (Fig 1). This low level of structure in our population reflected the diverse geographic origins of the apple cultivars included and strengthened the association analyses (Table 1) [35–36].

While all apple cultivars were considered to fall within the “dessert apple” market class [35–36], there was some separation of cultivars based on cultivar designation (heritage vs. commercial) along PC1 (Fig 1). This separation could reflect some differences in temporal origins. The heritage cultivars were mostly released before the 20^th^ century while the commercial cultivars were mostly released during the 20^th^ century and in some cases share common ancestors (Table 1) [35–36]. The shared ancestry of some commercial cultivars was reflected in the kinship analysis which revealed the population to be highly unrelated with a few pairs of more closely related genotypes (Fig 1). The potential confounding effects of population stratification were included as fixed effects in both GLM and MLM analyses to avoid the detection of spurious associations [39,58].

### Apple GWAS

The reference trait apple skin colour was mapped prior to GWAS of the fruit quality traits (Fig 3). The GLM analysis provided more statistical power to detect significant associations than MLM. A clear association signal on chromosome 9 was observed for skin colour using the MLM analysis, but this association fell below the 5% FDR threshold. The most significant locus for both analyses corresponded to the expected gene position of *MdMYB1* on chromosome 9 (Fig 3) [38,57].

While MLM is known to be a more robust tool for GWAS in populations for which familial relationships may cause spurious results [58], it is clear from the apple skin colour analysis that the increase in stringency of MLM can be an impediment to the detection of important sources of variation (Fig 3). The reduced power of detection using MLM has been discussed [58], and is demonstrated in a recent MLM GWAS conducted using 115 diverse apple accessions which was unable to detect any significant associations for the sensory traits “crispness,” “juiciness” or “flavour intensity” [22].

For the present study which made use of a moderate population size, we have reported the significant findings of GLM GWAS. The apple skin colour mapping results indicated that the apple population, GBS data set and GLM GWAS approach were sufficiently robust to detect known associations and were therefore appropriate to use in mapping fruit quality traits (Figs 3 and 4). Once significant associations were detected in GLM analyses, we compared the results with MLM plots to determine whether associations were still evident when kinship effects were taken into account (Figs 3 and 5). The sensory fruit quality loci we report are QTL which show evidence of coincident association in GLM and MLM GWAS, or are consistent with previous findings (Table 3).

### Apple taste and flavour

The sensory approach to apple phenotyping was chosen for its direct measurement of taste, flavour and texture traits as they will ultimately be perceived by consumers (Table 2). For example, sweet taste is known to be a preferred trait for consumers [5], but few MAS targets for sweetness have been proposed [64]. SSC is an instrumental measure commonly used to approximate the sugar content and predict the sweetness of apple fruits [32]. Many apple quality mapping studies have used SSC data for QTL discovery [64].

Previous studies of SSC and sugar content in apple have suggested that these traits are controlled by a large number of small effects loci [64]. Our results were not consistent with these previous studies, as a large percentage of variation (R^2^ = 0.43) was explained by the single significant locus we detected for SSC at Chr8:24,235,959 (Fig 4, Table 3). This result supported a QTL previously reported on chromosome 8 in the progeny of ‘Fiesta’ x ‘Discovery,’ which explained 11% of the SSC variation [23]. Although most studies of SSC including that by [23] have reported multiple QTL, a recent GWAS of 115 apple accessions did not find any significant SNPs [22]. Our result could be considered consistent with that of [22] in that the vast majority of SNP effects were scattered below the 5% FDR threshold (Fig 4). Further, we did not detect any significant associations for sensory *sweet* taste.

Since no direct associations were detected for *sweet* taste in this study, we examined the correlations between *sweet* and other fruit quality traits. *Sweet* taste was poorly correlated with SSC (ρ = 0.18, Fig 2), a result which has previously been reported [6,32,61]. Thus the Chr8:24,235,959 SSC locus was determined to be of little benefit to consumer focused apple breeding. However sweet taste was found to be negatively correlated with *fresh green apple* flavour (ρ = -0.62, Fig 2). *Fresh green apple* flavour was defined by the sensory panel as ‘grassy, vegetal aroma’ (Table 2). Apple flavour and aroma have previously been shown to influence the perception of sweetness and consumer preference [6,65]. This relationship between taste and flavour was particularly compelling to this study since two significant loci were detected for *fresh green apple* flavour (Fig 4, Table 3).

Previous studies of apple sensory flavour have detected significant associations with the major apple acidity loci on chromosomes 8 and 16 [66]. Although *fresh green apple* was positively correlated with TA (ρ = 0.58), neither acidity locus was significant in this study (Figs 2 and 4). Rather, the Chr9:4,092,040 and Chr12:10,115,943 *fresh green apple* flavour loci were both proximal to QTL for apple volatile compounds known to contribute to fruity apple aroma (Table 3) [65–68]. The co-localization of the *fresh green apple* loci with QTL for several apple aroma volatiles was consistent with previous suggestions that apple sensory flavour arises from the interaction of multiple volatile compounds detected simultaneously by the olfactory system [65].

The Chr9:4,092,040 and Chr12:10,115,943 fresh *green apple loci* present good potential targets for MAS. The loci each captured a moderate proportion of the variation in *fresh green apple* flavour, with 26% of the trait variation explained by Chr9:4,092,040 and 28% explained by Chr12:10,115,943 (Table 3). Both loci also has significant effects on apple sensory *acid* and *sweet* taste (S3 Table). Apple cultivars homozygous unfavourable for both loci such as ‘Granny Smith’ and ‘Ontario’ had high acidity and low sweetness phenotypes, and may therefore represent a candidate haplotype for deselection. The potential partially additive interaction between the Chr9:4,092,040 and Chr12:10,115,943 *fresh green apple* loci merits further investigation in a larger diverse population or segregating germplasm. If they can be validated in a larger population, then applying these *fresh green apple* loci in MAS would permit overall improvement in *sweet* taste as well as in a number of apple fruit quality traits for which significant associations were not detectible, due partly to poor correlations between years and low heritabilities (Fig 2).

### Apple texture

The GLM GWAS revealed four significant loci for sensory texture (Fig 4, Table 3). Three of these significant loci were positioned on the same chromosomes as previously reported QTL for apple texture, while the fourth was positioned on a chromosome for which no previous QTL have been reported (Table 3).

Chromosome 10 has been widely reported to contain a major QTL for apple texture which explains between 20 and 49% of the variation in acoustic-mechanical texture parameters [25,69]. In this study, one significant locus for *mealy* texture and one significant locus for *skin thickness* were detected on chromosome 10 (Fig 4, Table 3). These loci were separated by 3.7 Mb and were located toward the bottom of chromosome 10. Both loci captured a moderate proportion of the variation in their respective sensory texture traits, with Chr10:24,387,143 explaining 26% of the variation in *mealy* and Chr10:28,123,441 explaining 25% of the variation in *skin thickness* (Table 3). These results suggest an important role for chromosome 10 in the texture properties of apple fruit, which include both instrumental traits that can be precisely and systematically measured, as well as sensory traits which can be critical to predicting consumer preference [6,25,46].

The detection of significant sensory texture loci on chromosome 10 was also consistent with the findings of previous apple sensory QTL studies [10,26]. A moderate effect QTL was reported on chromosome 10 for the traits “slow breakdown” and “hardness” in a descriptive sensory evaluation study [10]. A large effect QTL on chromosome 10 was also reported for the trait “firmness” in a sensory texture study [26]. The trait “slow breakdown” was described similarly to *mealy* texture which our sensory panel defined as ‘the sensation of soft, granular flesh’ (Table 2) [10]. The traits “hardness” and “firmness” were described similarly to *skin thickness* which our panel defined as ‘the amount of force needed to bite through apple skin’ (Table 2) [10,26]. While the study by [10] used 11 trained sensory panelists and the study by [26] used four expert tasters, both studies reported an important chromosome 10 QTL for sensory apple texture, which our results support. Together, these results indicate that sensory phenotypic data can be used to validate QTL first detected in instrumental analyses, and that the chromosome 10 apple texture QTL is robust to different methods of sensory evaluation.

The genes *MdPG1* and *MdACO1*, which are located toward the centre and bottom of chromosome 10, respectively, are candidate genes controlling apple firmness and storability [13,15]. Although distal to the significant loci for *mealy* and *skin thickness* (Table 3) [38], these two genes may have contributed to textural variation in the apple population. The commercial cultivars included in this study were enriched for favourable alleles of both genes, and had phenotypes with lower *mealy* and *skin thickness* values (S4 Table). While the effects of chromosome 10 on sensory texture were moderate in this study, it is possible that larger effects would be detected after a longer period of storage as is commonly employed in apple texture mapping studies [25,26,69]. Conducting MAS for apple texture based on chromosome 10 would likely benefit more from using the well-characterized genomic variation in *MdPG1* and *MdACO1* than from the two chromosome 10 loci presented on this study. Neither Chr10:24,387,143 nor Chr10:28,123,441 had significant effects on instrumental FF for which no significant loci were detected (S3 Table).

Previous studies of apple texture have detected QTL for fruit firmness and crispness on chromosome 5. A moderate effect QTL was reported on chromosome 5 for sensory crispness and hardness in the ‘Prima’ x ‘Fiesta’ population [10]. QTL on chromosome 5 were also reported for several mechanical texture parameters in the cross of ‘Fuji’ x ‘Deleary’, explaining approximately 20% of the trait variation [25]. Our detection of a significant locus at Chr5:11,526,314 which explained 23% of the variation in each *crisp* and *mealy* texture was consistent with these findings (Fig 4, Table 3) but was inconsistent with a study of 27 pedigreed apple families in which chromosome 5 did not contribute to fruit texture [69]. As well, no strong candidate apple texture genes have been proposed on chromosome 5 [25]. The MLM analyses also did not indicate strong associations for *crisp* and *mealy* sensory texture at Chr5:11,526,314 (Table 3). The absence of a candidate gene and the weak MLM associations suggested that Chr5:11,529,314 should not be investigated further as a potential locus for MAS.

To our knowledge, QTL for apple texture have not previously been reported on chromosome 13. Manhattan plots derived from MLM analyses in a recent GWAS showed evidence of an association with sensory juiciness on chromosome 13, however this association was not significant and was therefore not discussed by the authors [22]. The study by [22] was conducted in an apple germplasm collection that represented more genotypic and phenotypic diversity than the bi-parental mapping populations in which most apple texture QTL have been discovered. Two previous studies of apple sensory *crisp* and *juicy* texture in bi-parental populations did not find QTL for either trait on chromosome 13 [10,26]. Both QTL studies used similar descriptions as our sensory panel which defined *crisp* texture as the ‘sound and sensation of breaking apart apple flesh in a single bite,’ and *juicy* texture as the ‘amount of liquid released from apple flesh by chewing’ (Table 2), but the QTL reported were not consistent between studies [10,26]. The difference in *crisp* and *juicy* QTL detected by each study suggests that mapping sensory texture can be sensitive to the population under investigation.

In our study a significant sensory texture locus at Chr13:6,049,060 explained 23% of the variation in *crisp* and 25% of the variation in *juicy* texture (Table 3). Our results support the association between sensory juiciness and chromosome 13 observed in diverse germplasm [22]. The associations we detected using GLM analyses for both *crisp* and *juicy* at Chr13:6,040,060 were coincident in MLM analyses, which supports the validity of this texture locus (Fig 5). We consider Chr13:6,049,060 to represent an important new candidate texture QTL that was derived from robust descriptive sensory evaluation and that captures the variation in diverse apple germplasm.

A putative apple PME gene (MDP000021997) was identified 214 kb downstream of the Chr13:6,049,060 locus (Fig 7) [38]. Although located slightly beyond the large region of localized LD on chromosome 13 (Fig 7), this PME gene could be considered a candidate gene for control of apple sensory texture. PME genes are conserved in multicellular plants and function predominantly in the de-methlyesterfication of cell wall polygalacturonans [59]. Other apple pectinesterase genes have been found to collocate with previously reported texture QTL [25], and a recent study of apple PME expression during fruit maturation suggested a major role for this protein family in determining mealy fruit texture [60].

Mealy texture in apple is the result of cells separating from one another at the middle lamella without breaking [24]. In contrast, crisp and juicy texture results from breaking open tightly adhered cells by mastication [24]. The putative function of MDP000021997 would be consistent with this cellular model of apple sensory texture, given that PMEs have been proposed to control the breakdown of intercellular adhesions both through the direct depolymerisation of middle lamella pectins and through the regulation of polygalacturonase activity [70]. While the MDP000021997 mRNA was found to be constitutively expressed in six sibling mealy and non-mealy apples [60], this finding does not exclude the possibility that allelic variation in this PME gene could confer differential fruit texture phenotypes.

The large proportion of variation in sensory texture explained by allele class at the Chr13:6,049,060 sensory texture locus makes it an attractive tool for MAS regardless of whether MDP000021997 is the causative gene (Fig 6). Seven putative genes of unknown function were detected within 50 kb of Chr13:6,049,060, any of which might also underlie the observed association with apple sensory texture (Fig 7). Significant improvements to breeding populations could be made through the deployment of a single marker for the Chr13:6,049,060 SNP which would capture the relevant genetic variation.

Selection for the homozygous favourable and heterozygous alleles would have the potential to increase average crispness by 10% (R^2^ = 0.34), increase juiciness by 7% (R^2^ = 0.40) and decrease mealiness by 4% (R^2^ = 0.29) (Fig 6, S3 Table). Many high quality commercial apples including ‘Ambrosia,’ ‘Honeycrisp’ and ‘Jazz’ are fixed for the homozygous favourable allele, but the marker would be very useful in culling progeny derived from intercrossing heterozygotes such as ‘Fuji,’ ‘Silken’ and ‘SweeTango.’ Apple cultivars fixed for the homozygous unfavourable allele are uncommon due to their high mealiness which is an undesirable trait in modern apples [5]. For example, ‘McIntosh’ was the only commercial cultivar of this study to fall into the homozygous unfavourable allele class. Deploying the Chr13:6,049,060 SNP marker to identify and cull mealy seedlings from new breeding populations would permit significant and efficient gains from selection for three critical attributes of sensory texture.

### Conclusion

In summary, MAS for fruit quality based on human sensory perception removes the ambiguity of modeling perceived fruit quality as a function of analytical traits and directly selects the quality attributes themselves. Here we have shown the discovery of new loci controlling critical components of apple sensory flavour and texture with direct relevance to breeding selection. The results of this study in apple demonstrate that such an approach is practical, and we believe that it can be broadly applied in horticulture plant breeding.

## Supporting information

S1 Fig Spearman rank correlations between all fruit quality traits. The trait “Designation” refers to apple cultivars as either heritage or commercial. Rank correlations were estimated by comparison of fruit quality trait values in 78 apple genotypes for two-year combined data.
(PDF)

S1 Table Linkage disequilibrium decay summary and SNP distribution by chromosome for 85 apple cultivars. (PDF)

S2 Table Phenotypic distributions of 78 apple cultivars evaluated for instrumental and sensory fruit quality in 2012 and 2013. (PDF)

S3 Table Variance statistics for allele class of SNPs associated with fruit quality traits. (PDF)

S4 Table Genotypes of apple cultivars at the *MdACO1* and *MdPG1* gene loci. Alleles are indicated as: “F” = homozygous favourable, “H” = heterozygous, “N” = homozygous non-favourable and “-” = unknown. Genotyping was based on *MdACO1* and *MdPG1* apple texture candidate gene studies [13,15].
(PDF)

S5 Table Polymorphism data of all SNPs in all apple accessions including chromosome location and physical location. (7Z)

## References

[1] Velasco R, Zharkikh A, Affourtit J, Dhingra A, Cestaro A, Kalyanaraman A, et al The genome of the domesticated apple (Malus x domestica Borkh.) Nat Genet 2011; 10.1038/ng.654 20802477

[2] Food and Agriculture Organization (FAO) of the United Nations. FAOSTAT database. 2016; Available: http://faostat3.fao.org/compare/

[3] Hancock JF, Luby JJ, Brown SK, Lobos GA. Temperate Fruit Crop Breeding: Germplasm to Genomics. Springer; 2008.

[4] Hampson CR, Quamme HA, Hall JW, MacDonald RA, King MC, Cliff MA. Sensory evaluation as a selection tool in apple breeding Euphytica 2000;

[5] Harker FR, Kupferman EU, Marin AB, Gunson FA, Triggs CM. Eating quality standards for apples based on consumer preferences. Postharvest Biol Tec 2008;

[6] Cliff MA, Stanich K, Lu R, Hampson CR. Use of descriptive analysis and preference mapping for early-stage assessment of new and established apples. J Sci Food Agr 2015; 10.1002/jsfa.7334 26171961

[7] Maliepaard C, Alston FH, van Arkel G, Brown LM, Chevreau E, Dunemann F, et al Aligning male and female linkage maps of apple (Malus pumila Mill.) using multi-allelic markers. Theor Appl Genet 1998;

[8] Xu K, Wang A, Brown S. Genetic characterization of the *Ma* locus with pH and titratable acidity in apple. Mol Breed 2011;

[9] Bai Y, Dougherty L, Li M, Fazio G, Cheng L, Xu K. A natural mutation-led truncation in one of the two aluminum-activated malate transporter-like genes at the Ma locus is associated with low fruit acidity in apple. Mol Genet Genomics 2012; 10.1007/s00438-012-0707-7 22806345

[10] King GJ, Maliepaard C, Lynn JR, Alston FH, Durel CE, Evans KM, et al Quantitative genetic analysis and comparison of physical and sensory descriptors relating to fruit flesh firmness in apple (*Malus pumila* Mill.) Theor Appl Genet 2000;

[11] Maliepaard C, Sillanpaa MJ, van Ooijen JW, Jansen RC, Arjas E. Bayesian versus frequentist analysis of multiple quantitative trait loci with an application to an outbred apple cross. Theor Appl Genet 2001;

[12] Kenis K, Keulemans J, Davey MW. Identification and stability of QTLs for fruit quality traits in apple. Tree Genet Genomes 2008;

[13] Costa F, Stella S, Van de Weg E, Guerra W, Cecchinel M, Dallavia J, et al Role of the genes *Md-ACO1* and *Md-ACS1* in ethylene production and shelf life of apple (*Malus domestica* Borkh). Euphytica 2005;

[14] Costa F, Van de Weg WE, Stella S, Dondini L, Pratesi D, Musacchi S, et al Map position and functional diversity of *Md-Exp7*, a new putative expansin gene associated with fruit softening in apple (*Malus* x *domestica* Borkh.) and pear (*Pyrus communis*). Tree Genet Genomes 2008;

[15] Costa F, Peace CP, Stella S, Serra S, Musacchi S, Bazzani M, et al QTL dynamics for fruit firmness and softening around an ethylene-dependent polygalacturonase gene in apple (*Malus* x *domestica* Borkh.). J Exp Bot 2001; 10.1093/jxb/erq130 PMC2892147 20462945

[16] Ru S, Main D, Evans K, Peace C. Current applications, challenges, and perspectives of marker-assisted seedling selection in Rosaceae tree fruit breeding. Tree Genet Genomes 2015;

[17] Luby JJ, Shaw DV. Does marker-assisted selection make dollars and sense in a fruit breeding program? Am J Hort Sci 2001; 36: 872–879.

[18] Baumgartner IO, Kellerhals M, Costa F, Dondini L, Pagliarani G, Gregori R et al Development of SNP-based assays for disease resistance and fruit quality traits in apple (*Malus* x domestica Borkh.) and validation in breeding pilot studies. Tree Genet Genomes 2016;

[19] Myles S, Peiffer J, Brown PJ, Ersoz ES, Zhang Z, Costich DE, et al Association mapping: critical considerations shift from genotyping to experimental design. Plant Cell 2009; 10.1105/tpc.109.068437 PMC2751942 19654263

[20] Khan MA, Korban SS. Association mapping in forest trees and fruit crops. J Exp Bot 2012; 10.1093/jxb/ers105 22511806

[21] Kumar S, Garrick DG, Bink MC, Whitworth C, Chagne D, Volz RK. Novel genomic approaches unravel genetic architecture of complex traits in apple. BMC Genomics 2013; 10.1186/1471-2164-14-393 PMC3686700 23758946

[22] Kumar S, Raulier P, Chagne D, Withworth C. Molecular-level and trait-level differentiation between the cultivated apple (*Malus* x *domestica* Borkh.) and its main progenitor *Malus sieversii*. Plant Genet Resour 2014;

[23] Liebhard R, Kellerhals M, Pfammatter W, Jertmini M, Gessler C. Mapping quantitative physiological traits in apple (*Malus* x *domestica* Borkh.) Plant Mol Biol 2003; 10.1023/a:1024886500979 12956523

[24] Harker FR, Hallett IC. Physiological changes associated with the development of mealiness of apple during cool storage. HortScience 1992; 27: 1291–1294.

[25] Longhi S, Moretto M, Viola R, Velasco R, Costa F. Comprehensive QTL mapping survey dissects the complex fruit texture physiology in apple (*Malus* x *domestica* Borkh). J Exp Bot 2012; 10.1093/jxb/err326 22121200

[26] Ben Sadok I, Tiecher A, Galvez-Lopez D, Lahaye M, Lasserre-Zuber P, Bruneau M, et al Apple fruit texture QTLs: year and cold storage effects on sensory and instrumental traits. Tree Genet Genomes 2015;

[27] Murray JM, Delahunty CM, Baxter IA. Descriptive sensory analysis: past, present, future. Food Res Intl 2001;

[28] Corollaro ML, Endrizzi I, Bertolini A, Aprea E, Dematte L, Costa F, et al Sensory profiling of apple: Methodological aspects, cultivar characterisation and postharvest changes. Postharvest Biol Tec 2013;

[29] Hoehn E, Gasser F, Guggenbuhl B, Kunsch U. Efficacy of instrumental measurements for determination of minimum requirements of firmness, soluble solids, and acidity of several apple varieties in comparison to consumer expectations. Postharvest Biol Tec 2003;

[30] Harker FR, Amos RL, Echeverria G, Gunson A. Influence of texture on taste: insights gained during studies of hardness, juiciness, and sweetness of apple fruit. J Food Sci 2006;

[31] Evans K, Brutcher L, Konishi B, Barrit B. Correlation of sensory analysis with physical and textural data from a computerized penetrometer in the Washington State University apple breeding program. HortTechnology 2010; 20: 1026–1029.

[32] Harker FR, Marsh KB, Young H, Murray SH, Gunson FA, Walker SB. Sensory interpretation of instrumental measurements 2: sweet and acid taste of apple fruit. Postharvest Biol Tec 2002;

[33] Stone H, Bleibaum R, Thomas HA. Sensory Evaluation Practices, 4^th^ Ed. Academic Press; 2012.

[34] Elshire RJ, Glaubitz JC, Sun Q, Poland JA, Kawamoto K, Buckler E. A robust, simple Genotyping-by-Sequencing (GBS) approach for high diversity species. PLoS ONE 2011; 10.1371/journal.pone.0019379 PMC3087801 21573248

[35] Beach SA. The Apples of New York Vol. II J.B. Lyon; 1905.

[36] Morgan J Richards A. The New Book of Apples. Elbury Press; 1993.

[37] Glaubitz JC, Casstevens TM, Lu F, Harriman J, Elshire RJ, Sun Q, et al TASSEL-GBS: A High Capacity Genotyping by Sequencing Analysis Pipeline. PLoS ONE 2014; 10.1371/journal.pone.0090346 PMC3938676 24587335

[38] Jung S, Ficklin SP, Lee T, Cheng CH, Blenda A, Zheng P, et al The Genome Database for Rosaceae (GDR): year 10 update. Nucleic Acids Res 2013; 10.1093/nar/gkt1012 PMC3965003 24225320

[39] Bradbury PJ, Zhang Z, Kroon DE, Casstevens TM, Ramdoss Y, Buckler ES. TASSEL: Software for association mapping of complex traits in diverse samples. Bioinformatics 2007; 10.1093/bioinformatics/btm308 17586829

[40] Gardner KM, Brown P, Cooke TF, Cann S, Costa F, Bustamante C, et al Fast and cost-effective genetic mapping in apple using next-generation sequencing. G3 2014; 10.1534/g3.114.011023 PMC4169160 25031181

[41] Money D, Gardner K, Migicovsky Z, Schwaninger H, Zhong GY, Myles S. LinkImpute: Fast and accurate genotype imputation for nonmodel organisms. G3 2015; 10.1534/g3.115.021667 PMC4632058 26377960

[42] Endelman JB, Jannink JL. Shrinkage estimation of the realized relationship matrix. G3 2012; 10.1534/g3.112.004259 PMC3484671 23173092

[43] Purcell S, Neale B, Todd-Brown K, Thomas L, Ferreira MA, Bender D, et al PLINK: a toolset for whole-genome association and population-based linkage analysis. Am J Hum Genet 2007; 10.1086/519795 PMC1950838 17701901

[44] Marroni F, Pinosio S, Zaina G, Fogolari F, Felice N, Cattonaro F, et al Nucleotide diversity and linkage disequilibrium in *Populus nigra* cinnamyl alcohol dehydrogenase (*CAD4*) gene. Tree Genet Genomes 2011;

[45] Blanpied GD, Silsby KJ. Predicting harvest date windows for apples. Cornell Cooperative Extension Publication: Information Bulletin 221; 1992.

[46] Harker FR, Maindonald J, Murray SH, Gunson FA, Hallet IC, Walker SB. Sensory interpretation of instrumental measurements 1: texture of apple fruit. Postharvest Biol Tec 2002;

[47] Merk HL, Yarnes SC, Van Deynze A, Tong N, Menda N, Mueller LA, et al Trait diversity and potential for selection indices based on variation among regionally adapted processing tomato germplasm. J Am Soc Hortic Sci 2012; 137: 427–437.

[48] Bates D, Maechler M, Bolker B, Walker S. Fitting linear mixed-effects models using lme4. Journal of Statistical Software 2015;

[49] R Core Team. R: A language and environment for statistical computing. 2015; Available: http://www.R-project.org/

[50] Merk HL. Estimating heritability and BLUPs for traits using tomato phenotypic data. Extension: Plant Breeding and Genomics. 2014; Available: http://articles.extension.org/pages/61006/estimating-heritability-and-blups-for-traits-using-tomato-phenotypic-data

[51] Harrell FE, Dupont C. Package ‘Hmisc’ version 3.17. 2016; Available: https://cran.r-project.org/web/packages/Hmisc/Hmisc.pdf

[52] Cattell RB. The scree test for the number of factors. Multivariate Behav Res 1966; 1: 245–276. doi: 10.1207/s15327906mbr0102_10 26828106 10.1207/s15327906mbr0102_10

[53] Benjamini Y, Hochberg Y. Controlling the false discovery rate: a practical and powerful approach to multiple testing. J R Stat Soc 1995; 57: 289–300.

[54] Turner SD. qqman: an R package for visualizing GWAS results using Q-Q and manhattan plots. *biorXiv* 2014;

[55] Wickham H. ggplot2: Elegant Graphics for Data Analysis. Springer-Verlag; 2009.

[56] Johnson M, Zaretskaya I, Raytselis Y, Merezhuk Y, McGinnis S, Madden TL. NCBI BLAST: a better web interface. Nucleic Acids Res 2012; 10.1093/nar/gkn201 PMC2447716 18440982

[57] Ban Y, Honda C, Hatsuyama Y, Igarashi M, Bessho H, Moriguchi T. Isolation and functional analysis of a MYB transcription factor gene that is a key regulator for the development of red coloration in apple skin. Plant Cell Physiol 2007; 10.1093/pcp/pcm066 17526919

[58] Yang J, Zaitlen NA, Goddard ME, Visscher PM, Price AL. Advantages and pitfalls in the application of mixed-model association methods. Nature Genet 2014; 10.1038/ng.2876 PMC3989144 24473328

[59] Micheli F. Pectin methylesterases: cell wall enzymes with important roles in plant physiology. Trends Plant Sci 2001; 10.1016/s1360-1385(01)02045-3 11544130

[60] Segonne SM, Bruneau M, Celton JM, Le Gall S, Francin-Allami M, Juchaux M, et al Multiscale investigation of mealiness in apple: an atypical role for a pectin methylesterase during fruit maturation. BMC Plant Biol 2014; 10.1186/s12870-014-0375-3 PMC4310206 25551767

[61] Oraguzie N, Alspach P, Volz R, Whitworth C, Ranatunga C, Weskett R, et al Postharvest assessment of fruit quality parameters in apple using both instruments and an expert panel. Postharvest Biol Tec 2009;

[62] Leforestier D, Ravon E, Muranty H, Cornille A, Lemaire C, Giraud T, et al Genomic basis of the differences between cider and dessert apple varieties. Evol Appl 2015; 10.1111/eva.12270 PMC4516418 26240603

[63] Micheletti D, Dettori MT, Micali S, Aramini V, Pachecho I, Da Silva Linge C, et al Whole-genome analysis of diversity and SNP-major gene association in peach germplasm. PLoS ONE 2015; 10.1371/journal.pone.0136803 PMC4564248 26352671

[64] Guan Y, Peace C, Rudell D, Verma S, Evans K. QTLs detected for individual sugars and soluble solids content in apple. Mol Breed 2015;

[65] Mehinagic E, Symoneaux R, Jourjon F, Prost C. Characterization of odor-active volatiles in apples: influence of cultivar and maturity stage. J Agric Food Chem 2006; 10.1021/jf052288n 16569061

[66] Kumar S, Rowan D, Hunt M, Chagne D, Withworth C, Soulyere E. Genome-wide scans reveal genetic architecture of apple flavour volatiles. Mol Breed 2015;

[67] Dunemann F, Ulrich D, Boudichevskaia A, Grafe G, Weber WE. QTL mapping of aroma compounds by headspace solid-phase microextraction gas chromatography in the apple progeny ‘Discovery’ x ‘Prima.’ Mol Breed 2009;

[68] Souleyre E, Chagne D, Chen X, Tomes S, Turner RM, Wang MY, et al The AAT1 locus is critical for the biosynthesis of esters contributing to ‘ripe apple’ flavour in ‘Royal Gala’ and ‘Granny Smith’ apples. Plant J 2014; 10.1111/tpj.12518 24661745

[69] Bink MC, Jansen J, Madduri M, Voorrips RE, Durel CE, Kouassi AB, et al Bayesian QTL analyses using pedigreed families of an outcrossing species, with application to fruit firmness in apple. Theor Appl Genet 2014; 10.1007/s00122-014-2281-3 24567047

[70] Brummell DA. Cell wall disassembly in ripening fruit. Funct Plant Biol 2006 10.1071/FP05234 32689218

