# Supplementary material for: Mapping the sensory perception of apple using descriptive sensory evaluation in a genome wide association study
Source: PLoS One. 2017 Feb 23;12(2):e0171710. doi: 10.1371/journal.pone.0171710 (PMC5322975; doi:10.1371/journal.pone.0171710)
Supplement: S1 Fig — The trait “Designation” refers to apple cultivars as either heritage or commercial. Rank correlations were estimated by comparison of fruit quality trait values in 78 apple genotypes for two-year combined data. (PDF) [file pone.0171710.s001.pdf]

S1 Fig. Spearman rank correlations between all fruit quality traits. The trait “Designation” refers to apple cultivars as either heritage or commercial. Rank correlations were estimated by comparison of fruit quality trait values in 78 apple genotypes for two-year combined data.

	Designation	Skin Colour	Flesh Firmness	Soluble Solids Content	Titratable Acidity	Acid	Bitter	Sweet	Earthy	Floral	Fresh Green Apple	Fresh Red Apple	Honey	Lemony	Oxidized Red Apple	Astringent	Chewy	Crisp	Juicy	Mealy	Rate of Melt	Skin Thickness
Designation	1.00																					
Skin Colour	0.11	1.00																				
Flesh Firmness	-0.11	-0.31	1.00																			
Soluble Solids Content	-0.23	0.10	-0.01	1.00																		
Titratable Acidity	-0.38	-0.19	-0.05	0.25	1.00																	
Acid	-0.40	-0.21	-0.02	-0.05	0.85	1.00																
Bitter	-0.24	-0.21	-0.02	-0.13	0.65	0.72	1.00															
Sweet	0.44	0.29	-0.04	0.18	-0.76	-0.93	-0.75	1.00														
Earthy	0.23	-0.23	-0.04	0.08	-0.19	-0.25	-0.05	0.30	1.00													
Floral	0.14	0.28	-0.10	0.06	-0.32	-0.36	-0.25	0.45	0.16	1.00												
Fresh Green Apple	-0.23	-0.26	0.14	-0.03	0.58	0.62	0.52	-0.62	-0.15	-0.39	1.00											
Fresh Red Apple	0.15	0.53	-0.23	0.16	-0.45	-0.49	-0.52	0.61	0.17	0.48	-0.54	1.00										
Honey	0.24	0.25	-0.03	0.18	-0.68	-0.76	-0.71	0.82	0.23	0.57	-0.65	0.60	1.00									
Lemony	-0.41	-0.21	-0.08	-0.06	0.76	0.86	0.66	-0.80	-0.20	-0.22	0.61	-0.40	-0.64	1.00								
Oxidized Red Apple	-0.15	0.11	-0.38	0.13	-0.10	-0.20	-0.15	0.19	0.30	0.31	-0.40	0.24	0.32	-0.07	1.00							
Astringent	-0.25	-0.28	0.07	-0.22	0.59	0.71	0.75	-0.75	-0.20	-0.22	0.46	-0.43	-0.60	0.68	-0.15	1.00						
Chewy	-0.15	-0.01	0.39	-0.30	-0.04	0.20	0.19	-0.26	-0.26	-0.24	0.22	-0.17	-0.16	0.06	-0.47	0.18	1.00					
Crisp	0.39	-0.01	0.46	-0.09	-0.15	-0.15	-0.12	0.24	0.02	0.02	0.18	0.02	0.04	-0.22	-0.67	-0.16	0.28	1.00				
Juicy	0.45	0.22	0.07	-0.25	-0.23	-0.17	-0.10	0.29	-0.05	0.05	0.08	0.17	0.07	-0.22	-0.52	-0.16	0.20	0.79	1.00			
Mealy	-0.45	-0.16	-0.18	0.15	0.23	0.21	0.16	-0.32	0.09	-0.08	-0.10	-0.15	-0.12	0.27	0.60	0.16	-0.17	-0.83	-0.93	1.00		
Rate of Melt	0.23	0.14	-0.63	0.05	-0.03	-0.17	-0.14	0.25	0.25	0.16	-0.22	0.14	0.15	-0.07	0.41	-0.27	-0.62	-0.29	-0.01	0.05	1.00	
Skin Thickness	-0.40	0.05	-0.08	-0.11	0.19	0.39	0.39	-0.45	-0.14	-0.17	0.30	-0.10	-0.27	0.36	-0.10	0.32	0.63	-0.19	-0.16	0.26	-0.32	1.00
				**Instrumental**			**Taste**					**Flavour**							**Texture**			
