# Supplementary material for: Mapping the sensory perception of apple using descriptive sensory evaluation in a genome wide association study
Source: PLoS One. 2017 Feb 23;12(2):e0171710. doi: 10.1371/journal.pone.0171710 (PMC5322975; doi:10.1371/journal.pone.0171710)
Supplement: S1 Table — (PDF) [file pone.0171710.s002.pdf]

S1 Table. Linkage disequilibrium decay summary and SNP distribution by chromosome for 85 apple cultivars.

Chromosome Number	LD decay (kb)[A]	Chromosme length (kb)	# of SNPs	Average distance between SNPs (kb)
1	75.0	29,390	2,575	11.4
2	39.5	36,519	3,712	9.8
3	48.8	34,035	3,060	11.1
4	68.7	23,056	2,421	9.5
5	51.7	31,643	3,325	9.5
6	61.5	25,525	2,325	11.0
7	128.2	26,629	2,599	10.2
8	21.5	29,751	2,720	10.9
9	25.8	33,775	3,673	9.2
10	34.8	33,492	3,908	8.6
11	38.8	35,328	3,461	10.2
12	44.1	31,722	3,141	10.1
13	122.0	34,925	3,382	10.3
14	61.4	29,326	2,450	12.0
15	74.7	47,455	4,636	10.2
16	82.6	20,870	2,228	9.4
17	13.5	24,926	2,824	8.8
**Average**	58.4	31,080	3,085	10.1

[A] LD decay extents were estimated at $r^2 = 0.2$. Chromosome length estimates were obtained from the apple reference genome *Malus* x *domestica* v3.0.a1.
