# Supplementary material for: Mapping the sensory perception of apple using descriptive sensory evaluation in a genome wide association study
Source: PLoS One. 2017 Feb 23;12(2):e0171710. doi: 10.1371/journal.pone.0171710 (PMC5322975; doi:10.1371/journal.pone.0171710)
Supplement: S2 Table — (PDF) [file pone.0171710.s003.pdf]

S2 Table. Phenotypic distributions of 78 apple cultivars evaluated for instrumental and sensory fruit quality in 2012 and 2013

Trait Type	Trait[A]	2012				2013				2-Year					
		Min	Max	Mean	SE	Min	Max	Mean	SE	Min	Max	Mean	SE	r_{year}	H^2
Instrumental	Flesh Firmness	1.1	8.2	5.0	0.71	2.7	4.6	3.5	0.13	2.0	6.1	4.2	0.75	0.41	0.87
	Soluble Solids Content	11.3	17.8	14.0	0.45	11.6	17.2	13.3	0.30	11.8	17.3	13.6	0.52	0.42	0.79
	Titratable Acidity	3.0	9.8	6.6	0.06	1.9	11.6	6.0	0.04	3.0	9.8	6.1	0.15	0.61	0.94
Sensory Taste	Acid	12.0	45.6	26.1	0.14	12.4	55.1	26.2	0.10	12.7	49.0	25.7	0.08	0.68	0.88
	Bitter	11.0	14.3	12.4	0.21	11.3	14.8	12.9	0.16	11.3	15.1	13.0	0.12	0.30	0.63
	Sweet	13.6	36.9	24.8	0.13	11.8	43.2	25.5	0.12	13.2	40.4	25.7	0.09	0.80	0.87
Sensory Flavour	Earthy	7.3	9.2	8.1	0.27	9.9	12.1	10.6	0.21	9.3	11.0	10.0	0.19	0.23	0.58
	Floral	8.4	11.7	9.8	0.27	8.8	15.6	11.2	0.21	9.2	13.1	11.0	0.17	0.31	0.62
	Fresh Green Apple	11.4	28.5	16.4	0.35	10.7	16.7	13.0	0.20	11.6	21.6	14.3	0.20	0.65	0.74
	Fresh Red Apple	9.9	38.7	21.7	0.19	18.7	25.8	22.2	0.08	18.0	24.8	21.5	0.09	0.37	0.37
	Honey	6.6	15.5	9.2	0.24	7.9	25.3	14.4	0.18	7.7	21.4	13.1	0.19	0.53	0.88
	Lemony	9.0	16.4	11.9	0.30	7.9	27.3	14.8	0.17	9.6	22.3	14.1	0.14	0.50	0.77
	Oxidized Red Apple	8.9	13.9	11.0	0.31	9.8	26.0	14.1	0.23	11.4	18.7	13.9	0.19	0.36	0.67
Sensory Texture	Astringent	11.1	17.3	14.0	0.37	17.7	13.0	15.1	0.24	13.0	17.3	14.9	0.18	0.38	0.68
	Chewy	34.7	47.2	41.5	0.13	27.8	63.6	47.8	0.08	38.7	52.0	45.6	0.08	0.36	0.59
	Crisp	14.2	46.3	31.7	0.21	15.0	70.0	44.7	0.09	15.7	58.6	39.1	0.12	0.78	0.92
	Juicy	18.6	51.8	36.5	0.11	21.9	66.3	46.6	0.05	22.1	56.5	41.9	0.09	0.75	0.88
	Mealy	13.8	51.8	22.6	0.25	11.2	50.1	18.6	0.24	14.2	52.5	22.1	0.23	0.71	0.88
	Rate of Melt	42.1	68.0	54.3	0.06	41.7	70.9	56.8	0.05	50.3	59.8	55.8	0.04	0.25	0.41
	Skin Thickness	46.7	55.2	51.0	0.10	36.1	67.1	53.3	0.06	38.4	62.8	51.9	0.05	0.57	0.88

[A] Traits were recorded in the units: Flesh Firmness (kg), Titratable Acidity (g malic acid L-1), Soluble Solids Content (% Brix) and Sensory Traits (Intensity: 0 to 100). The abbreviations are: r_{year} = Pearson correlation between trait values in 2012 versus 2013, H^2 = broad sense trait heritability, Min = minimum trait value by genotype, Max = maximum trait value by genotype and SE = standard error of the mean.
