# Supplementary material for: Mapping the sensory perception of apple using descriptive sensory evaluation in a genome wide association study
Source: PLoS One. 2017 Feb 23;12(2):e0171710. doi: 10.1371/journal.pone.0171710 (PMC5322975; doi:10.1371/journal.pone.0171710)
Supplement: S3 Table — (PDF) [file pone.0171710.s004.pdf]

S3 Table. Variance statistics for allele class of SNPs associated with fruit quality traits.

SNP Position	SNP Position	Probability	R^2	Allele Class	Mean	SE	Letter Group[A]
Chr9:4,092,040	Fresh Green Apple	<0.0001	0.41	AA	17	0.5	A
				CA	15	0.3	B
				CC	15	0.3	B
	Acid	0.0021	0.15	AA	33	2.8	A
				CA	30	1.9	A
				CC	28	1.7	A
	Sweet	0.0098	0.11	AA	23	1.9	A
				CA	24	1.3	A
				CC	25	1.2	A
Chr12:10,115,943	Fresh Green Apple	<0.0001	0.45	CC	18	0.6	A
				CG	14	0.3	B
				GG	15	0.3	B
	Acid	<0.0001	0.21	AA	38	3.2	A
				CA	25	1.9	B
				CC	29	1.5	B
	Sweet	<0.0001	0.22	AA	18	2.3	A
				CA	28	1.4	B
				CC	26	1.0	B
Chr10:24,387,143	Mealy	0.0076	0.09	CG	28	1.8	A
				GG	22	1.0	B
	Fruit Firmness	0.9918	<0.01	CG	4.2	0.15	A
				GG	4.2	0.08	B
Chr10:28,123,441	Skin Thickness	0.0372	0.08	CC	49	2.1	A
				CG	49	1.4	A
				GG	53	0.6	A
	Fruit Firmness	0.2165	0.04	CC	3.7	0.28	A
				CG	4.1	0.18	A
				GG	4.2	0.08	A
Chr13:6,049,060	Crisp	<0.0001	0.34	AA	42	1.6	A
				CA	44	1.3	A
				CC	29	2.1	B
	Juicy	<0.0001	0.40	AA	44	1.1	A
				CA	46	0.9	A
				CC	33	1.5	B
	Mealy	<0.0001	0.29	AA	22	1.3	A
				CA	20	1.1	A
				CC	32	1.8	B
	Fruit Firmness	0.0470	0.07	AA	4.0	0.12	A
				CA	4.3	0.10	A
				CC	4.0	0.16	A

[A] All differences between allele class means with different letter groups are significant according to a t-test adjusted for multiple means comparisons ($\alpha = 0.05$).
