# Supplementary material for: Mapping the sensory perception of apple using descriptive sensory evaluation in a genome wide association study
Source: PLoS One. 2017 Feb 23;12(2):e0171710. doi: 10.1371/journal.pone.0171710 (PMC5322975; doi:10.1371/journal.pone.0171710)
Supplement: S4 Table — Alleles are indicated as: “F” = homozygous favourable, “H” = heterozygous, “N” = homozygous non-favourable and “-” = unknown. Genotyping was based on MdACO1 and MdPG1 apple texture candidate gene studies [13,15]. (PDF) [file pone.0171710.s005.pdf]

S4 Table. Genotypes of apple cultivars at the *MdACO1* and *MdPG1* gene loci. Alleles are indicated as: “F” = homozygous favourable, “H” = heterozygous, “N” = homozygous non-favourable and “-” = unknown. Genotyping was based on *MdACO1* and *MdPG1* apple texture candidate gene studies [13,15].

Variety	Code	*MdACO1*	*MdPG1*	Variety	Code	*MdACO1*	*MdPG1*
Akane	AKA	N	H	Lodi	LOD	N	-
Ambrosia	AMB	H	-	Lubsk Queen	LUB	N	H
Antonovka	ANT	N	-	Macoun	MAC	N	N
Aurora Golden Gala™	AUR	-	F	McIntosh	MCI	N	N
Baldwin	BAL	-	-	McMahon	MCM	-	-
Blenheim Orange	BLE	N	N	Moscow Pear	MOS	-	-
Blue Pearmain	BLU	N	N	Moyer Heritage	MOY	-	-
Bottle Greening	BOT	N	-	Mutsu	MUT	N	N
Canada Red	CAN	H	-	Newtown Pippin	NEW	H	H
Chenango Strawberry	CHE	-	-	Nicola™	NIC	H	F
Colvert	COL	-	-	Northern Spy	NOS	N	-
Cortland	COR	N	N	Northern Spy	NOR	N	N
Cox's Orange Pippin	COX	N	H	NovaSpy	NOV	H	N
Creston	CRE	N	H	Ontario	ONT	N	N
CrimsonCrisp™	CRI	H	F	Pear Gold	PEA	H	F
Dawn Mac	DAW	H	N	Pink Lady™	PIN	N	F
Divine™	DIV	-	F	Pomme Grise	POM	N	H
Early Joe	EAR	H	-	Quinte	QUI	N	N
Elstar (Commercial)	ELC	N	H	Red Atlas	REA	-	-
Elstar (Heritage)	ELH	N	H	Red Delicious	RED	F	H
Empire	EMP	H	N	Red Prince	REP	-	-
Esopus Spitzenburg	SPI	H	F	Rome Beauty	ROM	N	N
Fameuse	FAM	N	-	Roxbury Russet	ROX	N	
Freedom	FRE	N	H	Royal Gala	ROY	H	H
Fuji	FUJ	F	F	Russet	RUS	N	-
Ginger Gold	GIN	N	-	Salish™	SAL	F	H
Golden Delicious	GOD	N	H	Silken	SIL	N	H
Golden Russet	GOL	N	N	Smitten	SMI	-	-
Granny Smith	GRA	N	F	Snow	SNO	-	-
Grimes Golden	GRI	N	H	Spartan	SPA	H	N
Haas	HAA	H	-	St Lawrence	STL	-	-
Heritage Gala	GAH	N	-	Summer Rambo	RAM	N	-
Honeycrisp	HON	N	F	SweeTango™	SWE	N	-
Hume	HUM	-	-	Tentation	TEN	N	F
Idared	IDA	H	H	Tolman Sweet	TOL	N	-
Irish Peach	IRI	N	-	Vinebrite	VIN	F	H
Jazz™	JAZ	-	F	Vista Bella	VIS	N	N
Jersey Mac	JER	N	N	Wealthy	WEA	H	F
Jonathan	JON	H	F	White Winter Calville	WHI	H	H
King	KIN	H	F	Winter Banana	WIN	N	N
Leder Borsdorf	LED	F	F	Yellow Bellflower	YEB	N	-
Liberty	LIB	H	H	Yellow Transparent	YET	N	N
Lobo	LOB	N	H				
